# Landscape heterogeneity, forest structure, and mammalian host diversity shape tick density and prevalence of the causative agent of Lyme borreliosis

**DOI:** 10.1093/femsec/fiaf088

**Published:** 2025-09-12

**Authors:** Sara Weilage, Max Müller, Lara Maria Inge Heyse, Dana Rüster, Manfred Ayasse, Martin Pfeffer, Anna Obiegala

**Affiliations:** Institute of Animal Hygiene and Veterinary Public Health, University of Leipzig, Leipzig, 04103, Germany; Institute of Evolutionary Ecology and Conservation Genomics, University of Ulm, Ulm, 89069, Germany; Institute of Ecological Networks, Technical University of Darmstadt, Darmstadt, 64287, Germany; Institute of Animal Hygiene and Veterinary Public Health, University of Leipzig, Leipzig, 04103, Germany; Institute of Animal Hygiene and Veterinary Public Health, University of Leipzig, Leipzig, 04103, Germany; Institute of Evolutionary Ecology and Conservation Genomics, University of Ulm, Ulm, 89069, Germany; Institute of Animal Hygiene and Veterinary Public Health, University of Leipzig, Leipzig, 04103, Germany; Institute of Animal Hygiene and Veterinary Public Health, University of Leipzig, Leipzig, 04103, Germany; Institute of Ecology and Environmental Science of Paris, Sorbonne University, Paris, 75005, France

**Keywords:** *Borrelia burgdorferi* s. l, forest structure, *Ixodes ricinus*, mammalian host diversity, One Health, tick-borne diseases

## Abstract

Ticks, particularly *Ixodes ricinus*, and the associated Lyme borreliosis risk, represent key concerns within the One Health framework, prompting extensive research in this field. However, comprehensive studies that jointly consider landscape characteristics, local forest structure and management, climate, and host community composition—alongside direct measures of tick density and infection status with *Borrelia* spp., the bacterial agents causing Lyme borreliosis, are scarce. In this study, we test the hypothesis that habitat diversity exerts a dilution effect, primarily by supporting greater diversity of mammal hosts. Therefore, we examined *I. ricinus* tick density and *Borrelia* spp. prevalence in relation to a comprehensive set of habitat and host-related variables. Ticks were collected using the flagging method and mammal hosts were monitored using an innovative camera-trapping approach across 25 forest plots along a land-use gradient within the Schwäbische Alb exploratory in Germany. Both tick density and *Borrelia* spp. prevalence are influenced by a complex combination of habitat factors across different spatial scales, as well as the mammal host community composition. Overall, our results provide novel support to the dilution effect hypothesis, suggesting that greater habitat and host diversity contribute to a reduced Lyme borreliosis risk in this region.

## Teaser

This study shows that diverse forests with rich mammal communities tend to have fewer infected ticks, helping to lower the risk of Lyme disease.

## Introduction

Ecological factors play a pivotal role in driving disease outbreaks by influencing the transmission dynamics of various zoonotic infectious diseases, such as Lyme borreliosis. Key organisms in the Lyme borreliosis (Europe) or Lyme disease (North America) transmission cycle always include a tick vector mainly of the genus *Ixodes* (e.g. *Ixodes ricinus*), a vector host (e.g. small or large mammals, birds) and a bacterial pathogen of the *Borrelia burgdorferi* sensu lato (s.l.) complex, which includes more than 20 different genospecies (Singh and Girschick 2004, Marques et al. 2021, Wolcott et al. 2021). In North America, a relatively well-defined host–vector–pathogen relationship, primarily involving the white-footed mouse *(Peromyscus leucopus)*, which serves as a highly competent reservoir host, *I. scapularis* ticks as the principal vector, and *B. burgdorferi* sensu stricto as the dominant human-pathogenic bacterial species, drives the Lyme disease risk. This core triad is central to pathogen transmission but there is a broader ecological context. White-tailed deer (*Odocoileus virginianus*), although not competent reservoirs for *Borrelia*, are key hosts for adult ticks and thus drive tick abundance and indirectly disease risk (Martin et al. 2023). Additionally, various small mammals (e.g. eastern chipmunks, short-tailed and masked shrews) and birds contribute to the maintenance and spread of *Borrelia* spp. across landscapes (Ginsberg et al. 2005, Levi et al. 2016). Despite this complexity, the North American system is still considered more ecologically constrained compared to the highly diverse host–vector–pathogen networks observed in Europe. This system involves a broad range of host species (including mammals, reptiles, and birds) across different trophic levels and ecosystems (Mannelli et al. 2012, Estrada-Peña et al. 2024). Further, Lyme borreliosis in Europe is caused by bacteria from the *B. burgdorferi* s.l. complex, consisting of at least 11 genospecies affecting multiple organ systems, including skin, heart, joints, and the nervous system, with varying severities of clinical outcomes in humans (Rizzoli et al. 2011, Trevisan et al. 2021). While host species are found in various ecosystems, the primary vector in Europe, *I. ricinus*, is known to have a high ecological plasticity, thriving in many habitat types and also at a range of altitudes (Kahl and Gray 2023). *Ixodes ricinus* is a three-host tick, meaning that each of its active developmental stages requires a separate blood meal from a vertebrate host to progress to the next stage. Its full life cycle, which typically spans 4–6 years, consists of egg, larval, nymphal, and adult (male or female) stages (Kahl and Gray 2023). *Ixodes ricinus* is also a major vector in Europe, capable of transmitting a wide range of zoonotic pathogens. This capacity is largely due to its broad host range, including over 300 vertebrate species, which are essential for the tick’s developmental life cycle (Gern and Humair 2002). Forest management, landscape configuration, climate, and host community composition all affect tick density and *Borrelia* spp. infection rates in ticks (Ehrmann et al. 2018, Bourdin et al. 2022, Król et al. 2022, Boulanger et al. 2024, Shaw et al. 2024). These factors are thus interdependent, yet they are rarely assessed collectively and can be challenging to quantify, especially in the complex European host–vector–pathogen system. The dilution effect hypothesis proposes that higher biodiversity reduces disease risk by decreasing the relative abundance of competent reservoir hosts. While many North American studies have provided empirical support for this concept (Allan et al. 2003, Keesing et al. 2006, LoGiudice et al. 2008), the overall evidence remains inconclusive. Results vary depending on ecological context, host community composition, and study design (Allan et al. 2003, Keesing and Ostfeld 2021). However, its applicability in more complex European systems remains uncertain. The greater host and pathogen diversity, coupled with varied forest management practices, complicates generalization. Indeed, European studies have yielded mixed (Gandy et al. 2022) or even contradictory (Ruyts et al. 2018) results, highlighting the need for more comprehensive approaches. Different groups of mammals have distinct functions in this predator–prey-host system and therefore have differing effects on the density of *I. ricinus* nymphs and *Borrelia* spp. prevalence (Occhibove et al. 2022). Most larger mammals are considered “dilution” or zooprophylactic hosts (see Table 1, *Capreolus capreolus, Lepus europaeus, Sus scrofa*, and *Sciurus vulgaris*) while most small mammals are considered reservoir hosts for *Borrelia* spp. Larger predatory mammals are considered “maintaining” hosts for *Borrelia* spp. but also exert top-down control on small mammal populations, which are important hosts for *I. ricinus* nymphs, by reducing their numbers and by altering their behavior through fear. When predator pressure is high, prey populations dwindle or spend less time foraging, which limits their exposure to questing ticks. Consequently, fewer ticks succeed in feeding and reproducing, and the prevalence of tick-borne pathogens declines since there are fewer reservoir hosts available (Hofmeester et al. 2017, Ruyts et al. 2018, Moll et al. 2020).

**Table 1.** Variables used in the regression models to determine nymph density and *Borrelia* spp. prevalence in nymphs.

Variable	Description	Unit	Datatype	Mean	SD
Broad leaf forest share	Proportion of broad leaf forest	%	**Landscape** (500 m radius) based on ATKIS data (Paul 2023)	0.2	0.2
H forest	Shannon’s diversity index of forest types (broad leaf, coniferous, mixed)	Index		0.8	0.2
Forest Cover	Proportion of forest cover calculated from all forest types	%		0.8	0.1
SMI	Silvicultural Management Index	Index	**Management** (Schall and Ammer 2024)	0.3	0.2
Tree species richness	Number of tree species per hectare = total tree species on the plot	Count (1/ha)	**Forest structure** (Schall and Ammer 2023, Hinderling and Prati 2024)	5.3	2.7
Crown projection area	Proportion of the forest floor that is covered by the vertical projection of the tree crowns	m^2^/ha		9709.6	1794.9
Mean tree dbh	Quadratic mean of the diameter at breast height of all trees with a diameter>7 cm on the plot	Cm		33.0	9.6
Dead wood volume	Sum of volume all lying dead wood with Ø>25 cm, length>1 m and all standing deadwood with Ø>25 cm, height>1.3 m	m^3^/ha		33.7	17.2
Shrub cover	Sum of each woody species cover with a height <5 m in relation to a 20 m × 20 m area on the plot.	%		23.0	20.6
Relative air humidity	Relative air humidity at 2 m height	%	**Climate** (Wöllauer *et al*. 2021)	83.9	3.2
RAI Predators	Number of events per 100 camera days of species predatory species (*Martes martes, Meles meles, Mustela putorius, Procyon lotor*, and *Vulpes vulpes)*	Index	**Mammal community** (mean of all seasons) (Müller 2025a, Müller 2025b)	56.7	64.1
H Predators	Shannon’s diversity index of predatory species (*Martes martes, Meles meles, Mustela putorius, Procyon lotor*, and *Vulpes vulpes)*	Index		0.7	0.2
RAI small mammals	Number of events per 100 camera days of small mammal species (*Apodemus flavicolis, Apodemus sylvaticus, Glis glis, Microtus arvalis, Muscardinus avellanarius, Myodes glareolus, Sorex araneus, Sorex minutus, Eraniceus europaeus*, and *Mustela nivalis)*	Index		2002.7	2355.3
H small mammals	Shannon’s diversity index of small mammal species (*Apodemus flavicolis, Apodemus sylvaticus, Apodemus sp., Glis glis, Microtus arvalis, Muscardinus avellanarius, Myodes glareolus, Sorex araneus, Sorex minutus, Eraniceus europaeus*, and *Mustela nivalis)*	Index		0.8	0.3
RAI large mammals	Number of events per 100 camera days of large non-predatory species (*Capreolus capreolus, Lepus europaeus, Sus scrofa*, and *Sciurus vulgaris*)	Index		166.8	69.4
H Large mammals	Shannon’s diversity index of large non-predatory species (*Capreolus capreolus, Lepus europaeus, Sus scrofa*, and *Sciurus vulgaris*)	Index		0.5	0.2
S total mammals	Total species richness of all mammals	Count		10.8	1.6
Nymph density	Number of *Ixodes ricinus* nymphs per m² per plot	Count	**Nymphs**	4.8	3.6
*Borrelia* prevalence	Number of *Borrelia*-positive nymphs/total number of nymphs collected	%		6.5	4.9

Landscape variables were calculated within a 500 m buffer around plot centers; local forest structure, management, and mammal diversity metrics were derived from data within the 1 ha plots.

The Swabian Alb in southwestern Germany exemplifies a region of high biodiversity and forest management gradients and thus offers an ideal setting to investigate these interactions. A previous study in the Swabian Alb suggested that on the one hand, forest management practices have an important impact on questing (i.e. actively host-seeking) *I. ricinus* nymph density but on the other hand that variable weather conditions may override these effects (Lauterbach et al. 2013). However, in the latter study, only a small size of individuals was sampled, the diversity of mammal hosts was not considered, and the ticks have not been tested for pathogens. Numerous other European studies have also examined the effects of specific variables on *I. ricinus* density or *Borrelia* prevalence (Ehrmann et al. 2018, Estrada-Peña et al. 2024, Olsthoorn et al. 2025). However, only a few have investigated the combined influence of habitat and host-related factors (Halos et al. 2010, Ruyts et al. 2018, Gandy et al. 2022, Boulanger et al. 2024), and these studies lack a fully comprehensive approach.

Drawing on this existing literature, we aimed to test the hypothesis that habitat diversity across different scales exerts a dilution effect, primarily by supporting greater diversity of mammal hosts, with small mammals playing a key role. To achieve this, we made use of the comprehensive set of habitat variables provided by the Biodiversity Exploratories database and supplemented it with targeted data collection of mammal hosts. Thus, to our knowledge, this is the first study to integrate landscape characteristics, forest structure and management intensity, climate, and mammal community host composition to assess their combined effects on *I. ricinus* density and *B. burgdorferi* s.l. prevalence in central Europe.

## Material and methods

### Study region

The Swabian Alb, located in the Reutlingen district of Baden-Wurttemberg, is one of three designated long-term research areas in Germany for biodiversity and ecosystem studies within the Biodiversity Exploratories program (Fischer et al. 2010). With an area of 420 km² it spans altitudes ranging from submontane to montane zones. The landscape is a patchwork of forests and open land, reflecting a variety of land-use practices. Since 2007, the Swabian Alb has been the focus of forest and grassland experiments, as well as extensive ecological observations. Data collected from the region, covering both biotic and abiotic factors, are accessible through an online platform and are integral to long-term scientific analysis (Chamanara et al. 2021).

**Figure 1.**
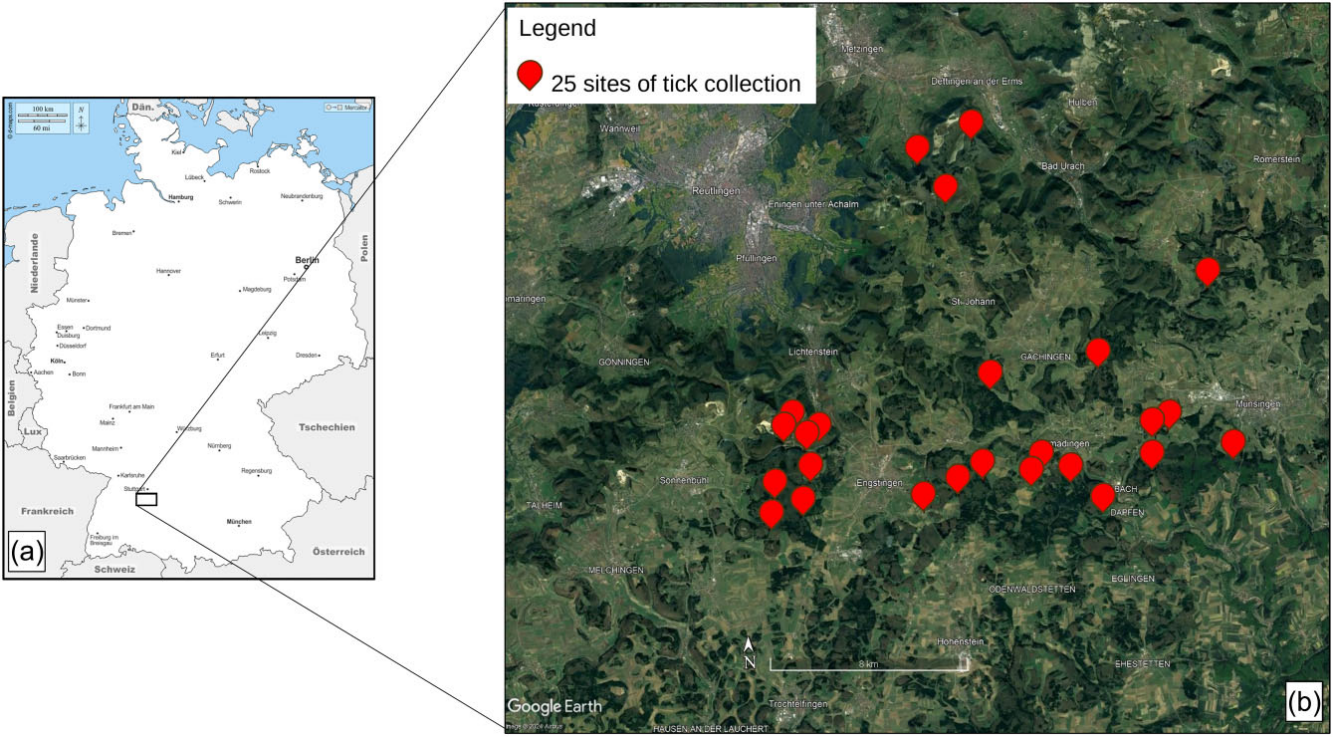
Geographical location of 25 tick collection sites (marked with red pins) along a land-use gradient in the Swabian Alb, Germany. Panel (a) shows the location of the study area in Germany and panel (b) shows the location of each plot. The image was created using Google Earth Pro, Map: Google Earth ^©^2025 Google, Image ^©^ 2025 Airbus, Image ^©^ 2025 Maxar Technologies, and https://d-maps.com/carte.php?num_car=2002&lang=de.

For this research, 25 of the 50 forest plots available in the Swabian Alb were chosen, representing the full range of the land-use gradient which was assessed using the Silvicultural Management Intensity Index (SMI), which evaluates key characteristics such as stand age, stand growth, and dominant tree species (Schall and Ammer 2013) (Fig. 1). In our case, lower SMI values indicate more extensively managed, beech-dominated forests, whereas higher SMI values correspond to more intensively managed stands dominated by conifers.

### Large mammal camera trapping

Two camera traps (Dörr Snapshot Mini Black 30 MP 4 K, sporadically predecessor models) were installed on each of the 25 plots according to a common scheme: height ∼50 cm, inclination of 5° downward, direction north, 3 pictures per trigger, 30 s non-reactive between triggers (Kammerle et al. 2018, Bubnicki et al. 2019). Two cameras per plot minimize site effects and prevent data gaps in the event of camera failure. Traps were installed at suitable locations enabling sufficient range and undisturbed triggering (Wening et al. 2019). Cameras were technically maintained at least every 6 months and relocated randomly to further minimize site effects. They were set up during the entire study from spring 2023 to spring 2024 with a total of 9609 camera days included in the analysis.

Processing of raw images and species identification was done using “Agouti”, an AI powered platform for managing wildlife camera trapping projects (Casaer et al. 2019). AI-based species identifications (AI tool “Western Europe v4a”) were subsequently confirmed through direct, in-person verification.

### Small mammal camera trapping

**Figure 2.**
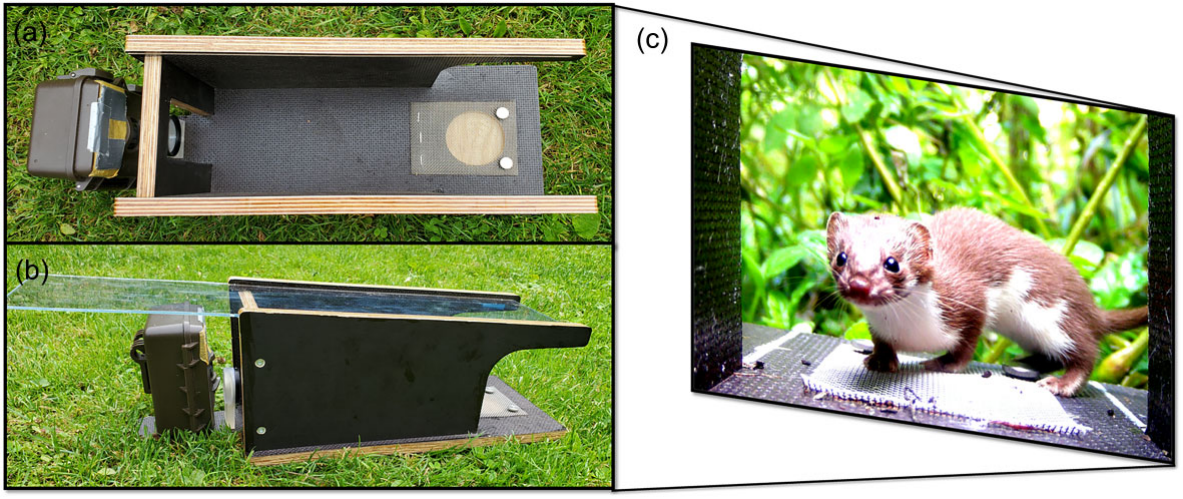
Small mammal camera trap adapted from Littlewood et al. (2021). Top and side view of Dörr Snapshot Mini Black 30 MP 4 K extended by a 4× magnifying lens, dimmed LED flash and box with bait under grid (a, b). Still image of a video (c, *Mustela nivalis*).

Custom-built small mammal camera traps, adapted from the design by Littlewood et al. (2021), were used for small mammal monitoring (Fig. 2). For this purpose, Dörr camera traps were equipped with a 4× close up-lens (dHD Digital). The flash unit was adjusted to a lower power level to prevent overexposure and attached to a box baited with a mixture of peanut butter, oats, and apples. The bait was placed out of reach under a grid to exclude a reward for the animal and to minimize frequent returns of the same individual. Small mammal camera trappings took place at the same location and during the same time periods as tick collection, as described below, in summer and autumn 2023 and in spring 2024. Two traps per each 300 m^2^ area were set up for 7 days per season, giving a total of 984 camera days. To facilitate species identification, traps were set to record 15 s videos at each trigger with 30 s delay before they could be triggered again. Species were identified according to nature field guides (Braun and Dieterlen 2003). In the case of *Apodemus*, species identification of *A. flavicollis* or *A. sylvaticus* was possible in 86% of the recordings. The remaining 14% were only identified to genus level and subsequently not used for Shannon diversity calculations.

### Tick collection, species identification and density calculation

In spring (May), summer (August), and autumn (October) of 2023, as well as in spring (May) of 2024, ticks were collected using the flagging method with a 100 × 100 cm white wool-cotton cloth. The cloth was slowly dragged over vegetation and leaf litter, and turned and inspected every 10 m to collect attached ticks and ensure consistent sampling efficiency. This standardized technique targets questing ticks and is widely used in ecological tick research (Estrada-Peña et al. 2013). The sampling area at each of the 25 previously mentioned plots measured 12 × 25 m and was permanently marked resulting in a total flagged area of 300 square meters per plot. All collected ticks were stored separately for each location at −20°C until further examination. Ticks were assigned to their developmental stage (larvae, nymph, adult male or female) and taxonomically identified to species level by using morphological keys (Estrada-Peña et al. 2017) under a stereomicroscope (Motic^®^ SMZ–171, Motic Europe, S.L.U., Barcelona, Spain). The nymphal tick density per 100 m^2^ was calculated for each location. Subsequent analyses were conducted exclusively on nymphal ticks.

### DNA extraction of ticks and molecular analyses for *Borrelia* spp.

To prepare DNA purification, 1 g of 2.8 mm steel beads (Peqlab Biotechnology, Erlangen, Germany) and 200 µl of phosphate-buffered saline (PBS) were added to each individual tick sample. Subsequently, each sample was homogenized using a Precellys^®^24 tissue homogenizer (Bertin Technologies, Montigny Le Bretonneux, France) at 5000 r/m for two cycles of 30 s each, with a 15-s pause between cycles. DNA extraction was performed using the QIAamp DNA Mini Kit (Qiagen Germany, Hilden) according to manufacturer’s instructions. The quality and quantity of DNA in each sample were assessed using a NanoDrop spectrophotometer (NanoDrop^®^ 2000c, Thermo Fisher Scientific, Waltham, MA, USA). The tick samples were screened for the presence of *Borrelia* spp. DNA by detecting the p41-flagellin gene (96 base pairs) through real-time polymerase chain reaction using the Mx3000 Real-Time Cycler (Agilent, Santa Clara, CA, USA) in accordance with an established protocol (Schwaiger et al. 2001).

### Statistical analysis

Relative abundance indices (RAIs) were calculated for each species as events per 100 camera days in R with the script described in Rovero and Zimmermann (2016):


\begin{eqnarray*}
RAI = \left( {\frac{{\textit{Number}\,\,of\,\,\textit{detections}\,\,for\,\,a\,\,\textit{species}}}{{\textit{Sampling}\,\,\textit{effort}\,\,\left( {\textit{camera}\,\,\textit{days}} \right)}}} \right)x\,\,100.
\end{eqnarray*}

The standardization of detections by the sampling effort makes the results comparable across time, space, and methods.

Shannon’s diversity index (H) was calculated from RAIs:


\begin{eqnarray*}
H = \,\,\mathop \sum \limits_i pi\,\,x\ln pi
\end{eqnarray*}

with $pi = \,\,\frac{{ni}}{N}$, *N* = total sum of RAIs, *n_i_* = sum of RAIs of respective species group.

We tested the effects of landscape variables, local forest management and structure, climate variables, and the mammal community on nymph density and *Borrelia* spp. prevalence in nymphs (Table 1). The mammal community was grouped in “large mammals” considered “dilution” or zooprophylactic hosts (see Table 1: *Capreolus capreolus, Lepus europaeus, Sus scrofa*, and *Sciurus vulgaris*), “small mammals” considered reservoir hosts for *Borrelia* spp. (see Table 1: *Apodemus flavicolis, Apodemus sylvaticus, Apodemus sp., Glis glis, Microtus arvalis, Muscardinus avellanarius, Myodes glareolus, Sorex araneus, Sorex minutus, Eraniceus europaeus*, and *Mustela nivalis*) and “predators” considered “maintaining” hosts for *Borrelia* spp. but also top-down-controller of small mammals (see Table 1: *Martes martes, Meles meles, Mustela putorius, Procyon lotor*, and *Vulpes Vulpes*). The landscape, management, structure, and climate variables were selected based on availability from the Exploratory data sets and their relevance to the study (Table 1, Ehrmann et al. 2018, Bourdin et al. 2022, Król et al. 2022, Boulanger et al. 2024).

For nymph density as response variable (right-skewed, not normally distributed), we fitted generalized linear models (GLMs) using a Gamma distribution with a logarithmic link function (S1, S6). For *Borrelia* spp. prevalence (proportional data, right-skewed with zero values) as response variable, we fitted GLMs with Tweedie distribution (S4, S8). Due to high collinearity (VIF > 10) air temperature at 2 m height, herb cover and conifer share on plot scale and forest edge density on landscape scale were excluded from all models. By comparison, relative air humidity at 2 m height, shrub cover, broadleaf share, and forest cover—which were collinear or even redundant with the excluded variables but considered ecologically more meaningful—were retained in the models. For *Borrelia* spp. prevalence, the initial full model exhibited signs of overfitting, multicollinearity (VIF > 10 for SMI and forest cover), and convergence issues (non-positive-definite Hessian matrix). To address this, the model was simplified by excluding variables with high collinearity. In addition, mean tree dbh and dead wood volume—both consistently non-significant in the full model and in a separate model including only the four excluded variables (data not shown)—were also removed. All statistical analyses were conducted in R version 4.3.1 (2023–06–16) (Team 2023). GLMs and LMs were run using the “glm” and “lm” function from the *stats* package, and the “glmTMB” from the *glmmTMB* package (Brooks et al. 2017). We selected and averaged the best fitting models (ΔAICc < 2), using the MuMln package (Barton 2020). We report conditional averaged model outputs and the full set of candidate models are provided in the Supplementary Information. For the seasonal analysis, best models are reported for nymph density as model dredging returned only single top-ranked models (AICc ≤ 2.0). In the seasonal models, total species richness had to be excluded due to high collinearity (VIF > 10).

### Data availability

This work is based on data elaborated by the Biodiversity Exploratories program (DFG Priority Program 1374). The datasets are publicly available in the Biodiversity Exploratories Information System (http://doi.org/10.17616/R32P9Q). The datasets are listed in the references section.

However, to give data owners and collectors time to perform their analysis the Biodiversity Exploratories’ data and publication policy includes by default an embargo period of 3 years from the end of data collection/data assembly which applies to the remaining datasets [IDs: 32109, 32111, 32113 (Müller 2025a, Müller 2025b, Weilage 2025)]. These datasets will be made publicly available via the same data repository.

## Results

### Tick collection

A total of 1816 ticks were collected. Two individuals could not be assigned to species level due to their poor conservation status, but all other collected ticks were identified as *I. ricinus* (*n* = 1814). Nymphs represented the most abundant life stage, with 1439 individuals (79.24%; CI 77.32–81.04), followed by larvae (*n* = 266; 14.65%; CI 13.09–16.35), females (*n* = 63; 3.47%; CI 2.72–4.42), and males (*n* = 48; 2.64%; CI 1.99–3.49) (Table 2, S1). Most nymphal ticks were collected in spring 2024 (*n* = 462; 32.11%; CI 29.69–34.52) followed by summer 2023 (*n* = 385; 26.75%; CI 24.47–29.04), spring 2023 (*n* = 338; 23.49%; CI 21.14–25.64), and autumn 2023 (*n* = 254; 17.65%; CI) (Table 2).

**Table 2.** Species, developmental stage, and number of collected ticks per season and in total.

Season and year	Tick species and developmental stage (number per season, *n* (%; CI 95%)					
		*Ixodes ricinus*				*Ixodes* spp.
	Total	Larva	Nymphs	Female	Male	Nymphs
Spring 2023	369	**1** (0.27; <0.01–1.68)	**338** (91.60; 88.29–94.05)	**23** (6.23; 4.15–9.22)	**7** (1.90; 0.84–3.94)	**0**
Summer 2023	467	**45** (9.63; 7.26–12.67)	**384** (82.24; 78.49–85.44)	**20** (4.28; 2.75–6.56)	**17** (3.64; 2.24–5.79)	**1** (0.21; 0.0054–1.19)
Autumn 2023	329	**58** (17.63; 13.88–22.13)	**253** (76.90; 72.04–81.14)	**13** (3.95; 2.26–6.71)	**4** (1.22; 0.36–3.20)	**1** (0.30;0.0077–1.68)
Spring 2024	651	**162** (24.88; 21.71–28.35)	**462** (70.97; 67.36–74.33)	**7** (1.08; 0.47–2.25)	**20** (3.07; 1.97–4.73)	**0**
**Total**	1816	**266** (14.64; 13.09–16.92)	**1437** (79.13; 77.20–80.94)	**63** (3.47; 2.72–4.42)	**48** (2.64; 1.99–3.49)	**2** (0.11; <0.01–0.43)

*n* = collected ticks [%; confidence interval (CI 95%)].

Only nymphal developmental stages were included in the analyses of *Borrelia* spp. prevalence, as they are generally considered the most relevant developmental stage for studying *Borrelia* transmission risk, due to their combination of relatively high abundance, infection prevalence, and being the most common stage feeding on human hosts (Table 3, S1, van Duijvendijk et al. 2016, Estrada-Peña et al. 2018, Kubiak et al. 2022).

**Table 3.** *Borrelia burgdorferi* sensu lato prevalence in all tested nymphal *I. ricinus* ticks among the different seasons and in total.

Season and year	*Borrelia burgdorferi* sensu lato prevalence
Spring 2023	**16/338** (4.73; 2.73–7.57)
Summer 2023	**22/385** (5.71; 3.63–8.51)
Autumn 2023	**24/254** (9.45; 6.61–13.74)
Spring 2024	**30/460** (6.52; 4.44–9.24)
**Total**	**92/1437** (6.40; 5.19–7.82)

*n* = positive ticks/ tested ticks [%; confidence interval (CI 95%)].

### Camera trapping

We recorded 5438 large mammal events of four non-predatory and five predatory species over 9609 camera days on the 25 plots from spring to autumn 2023 and in spring 2024 (Table 1, Müller 2025b). In summer and autumn 2023 as well as in spring 2024, 4626 small mammal events of 10 species were collected in a total of 984 camera days on the same 25 plots (Table 1, Müller 2025a). These mammal community data were combined with the silvicultural management index (SMI), local forest structure attributes, landscape characteristics, and climate variables to test the dilution effect hypothesis (Table 1).

### Ecological determinants of nymph density

**Figure 3.**
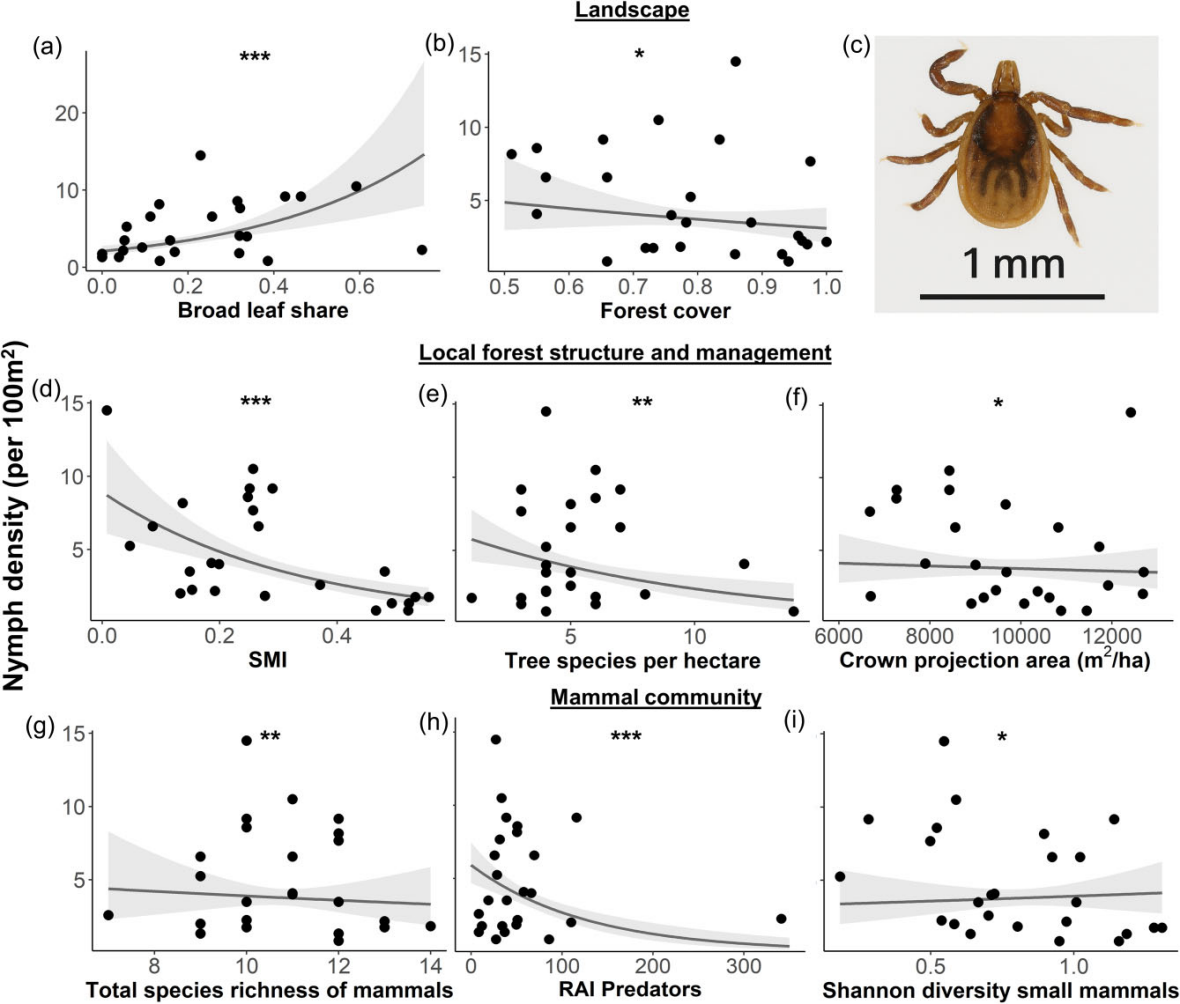
Ecological determinants of nymph density. Nymph density (per 100 m^2^) on 25 forest plots in the Swabian Alb in relation to significant factors of the conditional averaged generalized linear regression model using a Gamma distribution with a logarithmic link function: landscape factors (broad leaf share, forest cover, a, b), local forest structure and management (silvicultural management index (SMI), tree species per hectare, crown projection area, d, e, f), and mammal community (total species richness, relative abundance index (RAI) Predators, Shannon’s diversity index small mammals, g, h, i). Scatter points represent data at each forest plot. Lines represent fitted model curves. Shaded areas represent 95% confidence intervals. *P ≤ 0.05, **P ≤ 0.01, ***P ≤ 0.001. 10× magnified picture of an *I. ricinus* nymph (c).

Nymphal tick density strongly varied across the 25 forest plots, ranging from 0.00 to 46.67 individuals per 100 m^2^. The conditional average of the generalized linear regression using a Gamma distribution with a logarithmic link function retained nine explanatory variables, with eight statistically significant effects (Tables S2, S3, and Fig. 3). The density of nymphs was influenced by a combination of landscape characteristics, local forest structure and management practices, as well as the composition of the mammal community.

At the landscape scale, nymph density significantly decreased with a lower proportion of broadleaf forest (*z* = 4.265, *P* < 0.001) and with increasing forest cover (*z* = 2.201, *P* = 0.028, Fig. 3a, b). At the local plot scale, higher values of the SMI (*z* = 4.642, *P* < 0.001), greater tree species richness (*z* = 3.021, *P* = 0.003), and—albeit with minimal observable effect within the studied range—a larger crown projection area were all associated with a reduction in nymph density (*z* = 0.017, *P* = 0.017, Fig. 3d, e, f). Regarding mammal community, the RAI of predatory mammals was linked to a decrease in nymph density (*z* = 4.557, *P* < 0.001), while total species richness also contributed to this reduction, albeit with a small observable effect (*z* = 3.187, *P* = 0.001, Fig. 3g, h). The RAI of small mammals showed no evidence of a significant influence on nymph density in this analysis, whereas Shannon diversity exerted a small but significant positive effect (*z* = 2.386, *P* = 0.017, Fig. 3i).

### Ecological determinants of *Borrelia* prevalence in nymphs

A total of 1437 nymphal ticks were screened for *Borrelia* spp. Two individuals were excluded from testing as they were used for other analyses. Among the tested ticks, *Borrelia* spp. DNA was detected in 96 specimens; however, applying a cycle threshold (CT) of 41.42 reduced the number of confirmed positive cases to 92, resulting in an overall *Borrelia* prevalence of 6.4% (CI 5.19–7.82). Prevalence varied substantially across plots, ranging from 0.00% to 19.23%.

**Figure 4.**
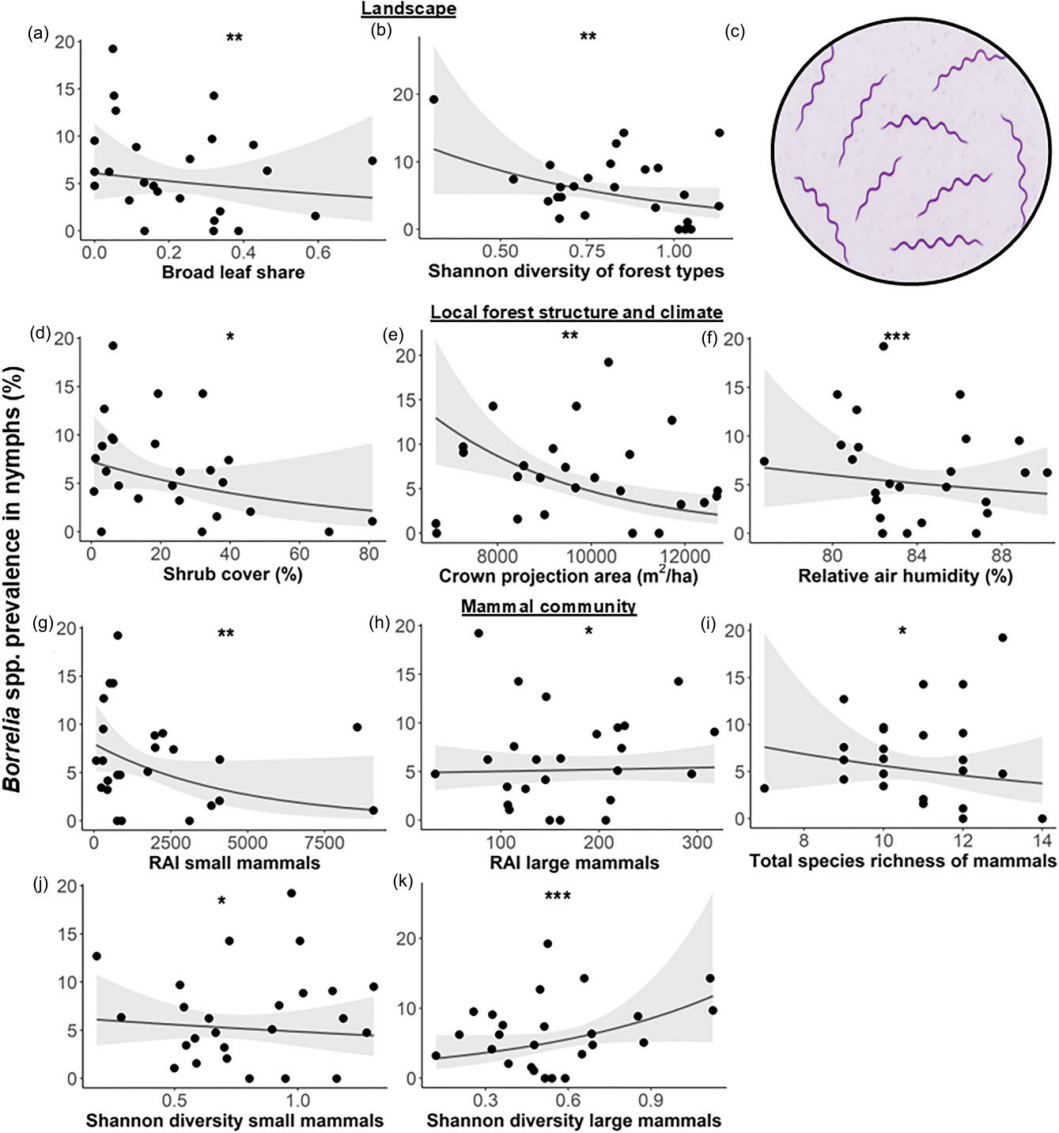
Ecological and climatic determinants of *Borrelia* prevalence in nymphs. *Borrelia* spp. prevalence in nymphs on 25 forest plots in the Swabian Alb in relation to significant factors of the conditional averaged generalized linear regression model using a Tweedie distribution: landscape factors (broad leaf share, Shannon diversity of forest types, (a, b), local forest structure and climate (shrub cover, crown projection area, relative air humidity, d, e, f), and mammal community [relative abundance index (RAI) of small and large non-predatory mammals, total species richness (S), Shannon diversity of small and large non-predatory mammals, g–k]. Scatter points represent data at each forest plot. Lines represent fitted model curves. Shaded areas represent 95% confidence intervals. *P ≤ 0.05, **P ≤ 0.01, ***P ≤ 0.001. (c) Schematic representation of *Borrelia*.

The conditional averaged generalized linear regression using a Tweedie distribution retained 10 explanatory variables with statistically significant effects indicating that *Borrelia* prevalence in nymphs is shaped by a multifactorial combination of landscape characteristics, local forest structure, climatic factors, and the mammal community (S4, S5, Fig. 4).

At the landscape scale, *Borrelia* spp. prevalence decreased with a higher proportion of broad-leaved forest (*z* = 2.671, *P* = 0.008) and greater diversity of forest types (*z* = 2.784, *P* = 0.006, Fig. 4a, b). On a local scale, higher shrub cover (*z* = 2.171, *P* = 0.030), larger crown projection area (*z* = 2.817, *P* = 0.005), and elevated relative air humidity (*z* = 2.881, *P* = 0.004) were all associated with lower *Borrelia* prevalence (Fig. 4d, e, f). Within the mammal community, greater total species richness (*z* = 2.426, *P* = 0.017), higher RAI of small mammals (*z* = 2.841, *P* = 0.004) and—albeit with minimal observable effect—higher Shannon diversity of small mammals (*z* = 2.381, *P* = 0.017) reduced prevalence (Fig. 4g, j, i). However, the Shannon diversity of large non-predatory mammals (*z* = 3.319, *P* < 0.001) and—albeit also with minimal observable effect—their RAI (*z* = 1.982, *P* = 0.048) had a positive effect on *Borrelia* prevalence (*P* = 0.045, Fig. 4h, k).

### Seasonal effects of the mammal community on nymph density

Since the small mammal population is subject to significant fluctuations, a seasonal analysis of the data was carried out. The development of nymphs after their blood meal as larvae takes ∼3 months, making the influence of the mammal population from the preceding season relevant for nymph density and infection risk. To account for this, we analyzed mammal community data collected in one season in relation to nymph density and *Borrelia* spp. prevalence in the subsequent season. Specifically, we examined summer mammal data alongside autumn tick data, representing directly consecutive seasons, and autumn mammal data with spring tick data, spanning the winter period. Nymphal tick densities were ranging from 0.67 to 15.67 per 100 m^2^ in autumn and 0.33 to 46.67 per 100 m^2^ in spring.

**Figure 5.**
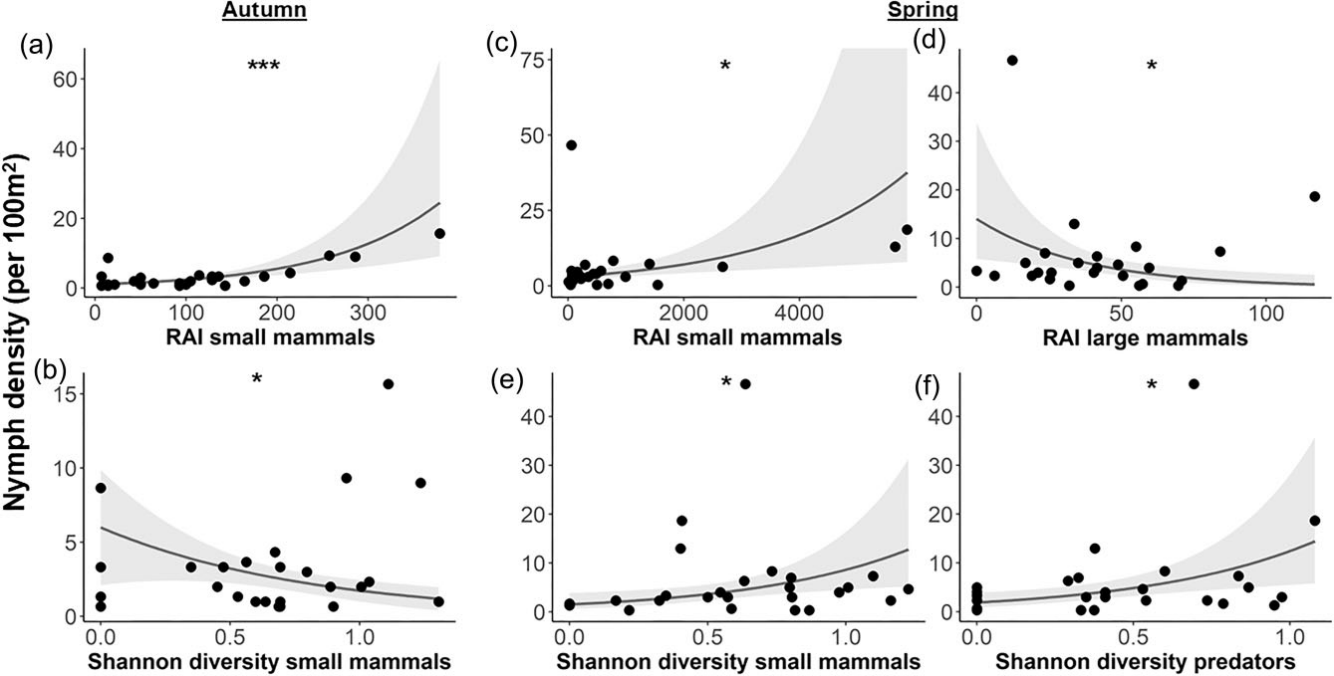
Determinants of the mammal host community for seasonal nymph densities in autumn and spring. Nymph density (per 100 m^2^) on 25 forest plots in the Swabian Alb in relation to significant factors of the best generalized linear regression model using a Gamma distribution with a logarithmic link function in autumn 2023 (a, b) and in spring 2024 (c–f). Scatter points represent data at each forest plot. Lines represent fitted model curves. Shaded areas represent 95% confidence intervals. Confidence intervals exceeding 100 were truncated for display purposes. *P ≤ 0.05, **P ≤ 0.01, ***P ≤ 0.001.

The best-fitting model retained two explanatory variables for the nymph density in autumn and four variables in spring, respectively. In both seasons, density of nymphs was influenced by a combination of mammal host abundance and diversity (S6, S7, Fig. 5).

As hypothesized, nymph density showed a positive relationship with the relative abundance of small mammals in both autumn (*t* = 4.583, *P* < 0.001) and spring (*t* = 2.768, *P* = 0.012, Fig. 5a, c). However, the influence of small mammal diversity differed between the two seasons, showing opposing trends: a negative association in autumn (*t* = −2.683, *P* = 0.014) and a positive association in spring (*t* = 2.541, *P* = 0.020, Fig. 5b, e). Additionally, nymph density in spring was negatively associated with the relative abundance of large mammals (*t* = −2.832, *P* = 0.010) and positively correlated with predator diversity (*t* = 2.806, *P* = 0.011, Fig. 5d, f).

### Seasonal effects of the mammal community on *Borrelia* prevalence in nymphs

A total of 24 nymphs tested positive for *Borrelia* spp. in autumn 2023 and 30 in spring 2024 (Table 3). The highest prevalence was recorded at a single sampling site in autumn 2023 with 50.00% and in spring 2024 with 27.27% (S1).

The conditional averages retained only one explanatory variable for *Borrelia* spp. prevalence in autumn and three variables in spring. However, the *Borrelia* spp. prevalence in nymphs did not appear to be significantly influenced by the mammal community in either season (S8, S9).

## Discussion

Our study takes an integrated approach to understand the ecological drivers of *I. ricinus* density and *B. burgdorferi* s.l. prevalence by combining landscape variables, forest structure, climate, and mammal community data within a single predictive model. Unlike previous studies that mostly examine these drivers in isolation, our approach reveals complex, scale-dependent combination shaping tick-borne disease risk (Halos et al. 2010, Ehrmann et al. 2018, Ruyts et al. 2018, Gandy et al. 2022, Boulanger et al. 2024, Estrada-Peña et al. 2024, Olsthoorn et al. 2025). Our findings support a nuanced view of the dilution effect—a hypothesis empirically supported in simpler North American systems—but still debated in the context of Europe’s more complex host communities (Ruyts et al. 2016, Ruyts et al. 2018, Keesing and Ostfeld 2021, Gandy et al. 2022). We show that while greater mammal species richness and a higher relative abundance of small mammals reduce *Borrelia* prevalence, increased diversity of large non-predatory mammals amplifies it.

### Landscape drivers

At the landscape scale (500 m buffer), we identified variables that reduce both tick density and *Borrelia* prevalence. A lower proportion of broadleaf forest was associated with a decrease in nymph density, likely due to the loss of favorable microhabitat conditions such as high humidity and dense leaf litter, which support tick survival and activity and reduced presence of small mammal hosts that thrive in such environments (Tack et al. 2012b, Ruyts et al. 2018, Zajac et al. 2021). In contrast, a higher proportion of broadleaf forest led to a decrease in *Borrelia* spp. prevalence. Previously, similar results have been observed for *B. afzelii—*a small mammal-associated genospecies, potentially due to a more diverse host community in broadleaf environments, which disrupt pathogen transmission cycles by increasing the presence of non-competent reservoir hosts. This pattern is consistent with the dilution effect hypothesis (Ruyts et al. 2016, Hofmeester et al. 2017, Keesing and Ostfeld 2021). Along the same lines, our results show that a greater forest-type diversity—reflecting landscape heterogeneity—was linked to lower *Borrelia* spp. prevalence. A similar pattern was also observed experimentally by Bourdin et al. (2022), though at smaller landscape scales.

In contrast to previous literature, forest cover showed a weak negative effect on overall nymph density but had no significant influence on *Borrelia* prevalence (Zajac et al. 2021). Similarly, forest edge density—previously associated with higher tick densities and *Borrelia* spp. prevalence (Tack et al. 2012a , Ehrmann et al. 2018, Boulanger et al. 2024)—had no effects in our models when used as predictor instead of the highly collinear forest cover (data not shown). Generalization of landscape effects should be interpreted with caution, as variable selection across spatial scales can yield differing or even opposing results (Ehrmann et al. 2018). Moreover, our survey plot was relatively small (300 m²), suggesting that broader landscape features may be less influential than fine-scale microhabitat conditions in shaping local tick density and *Borrelia* spp. prevalence.

### Local drivers

At the plot scale, several local forest habitat characteristics were associated with reductions in both *I. ricinus* nymph density and *Borrelia* prevalence. We show that a higher SMI, reflecting more intensive forest use and higher conifer share, is associated with lower nymph density. This is consistent with previous findings that conifer-dominated and intensively managed forests generally support fewer ticks (Tack et al. 2012b, Schall and Ammer 2013, Olsthoorn et al. 2025). Interestingly, an earlier 3-year study on tick density, conducted in the same plots within the Swabian Alb, found highest tick densities in highly managed young stands (Lauterbach et al. 2013). However, the study also highlighted the interannual variability in the predictive strength of environmental covariates and intriguingly a comparison of the average tick density on the same 25 plots ∼15 years later revealed no correlation whatsoever between past and present tick densities (*r*^2^ = 0.003, data not shown). In further contrast to previous studies, we found no direct effect of forest management intensity on *Borrelia* spp. prevalence (Olsthoorn et al. 2025).

The number of tree species per hectare was negatively associated with nymph density in our study, suggesting that increased tree diversity may reduce tick abundance by modifying microclimatic conditions or altering host communities—possibly through more complex food webs that decrease the availability of key tick hosts (Kahl and Gray 2023, Vacek et al. 2023).

Interestingly, crown projection area, indicative of older stands with denser canopies and typically higher humidity, was negatively associated with both nymph density—albeit with a small effect—and *Borrelia* spp. prevalence. This contrasts the common assumption that closed canopies benefit ticks by enhancing humidity—a factor that, notably, did not show any effect on nymph density in our study (Tukahirwa 1976), but it aligns with findings by Lauterbach et al. (2013), who observed higher nymph densities in younger stands. Furthermore, it suggests that the tick density-reducing effect of higher SMI is not primarily driven by tree felling and the resulting canopy opening. Greater shrub cover was another factor associated with lower *Borrelia* spp. prevalence, supposedly by enhancing habitats for a more diverse mammal community (Ruyts et al. 2016, Ehrmann et al. 2018). Overall, we assume that many landscape and forest management effects discussed thus far are indirectly mediated by changes in the mammalian host community.

### Mammal host community drivers

Indeed, the mammal host community composition plays a crucial role in regulating tick density and *Borrelia* spp. prevalence, in our study. Deer are regarded as essential for maintaining adult *I. ricinus* populations, while small mammals, such as *Apodemus spp*. and *Myodes glareolus*, sustain larval stages (Hofmeester et al. 2017). It should be noted that most *Borrelia* genospecies exhibit host specificity. While *B. garinii* and *B. valaisiana* are found in birds, *B. afzelii* is harboured by rodents, and *B. burgdorferi* sensu stricto is associated with both host groups (Margos et al. 2019). This underlines the need for future research to include genospecies-level identification in relation to host community composition, particularly in complex European ecosystems with a broad range of reservoir hosts. We used camera trapping as an effective, non-invasive method for assessing mammal communities (Rovero and Zimmermann 2016). While this method may introduce bias due to repeated sightings of the same individuals, the data also reflects host movement and activity, which are key determinants of tick encounter rates (Hofmeester et al. 2017).

We show that overall mammal species richness reduces nymph density and substantially lowers *Borrelia spp*. prevalence. Thus, our findings support the dilution effect hypothesis, suggesting that in the studied forest system higher mammalian species richness is associated with a decrease in the proportion of competent hosts (Keesing et al. 2006, Ogden and Tsao 2009). Distinguishing between predators and prey—and among prey, between large and small species—provided more nuanced insights into the factors influencing both *I. ricinus* nymph density and *Borrelia* spp. prevalence. Large non-predatory mammals, despite including roe deer (76.88% of this group) and Leporidae (5.96%)—widely acknowledged as multipliers of tick populations—did not significantly impact nymph density in our model (Hofmeester et al. 2017, Boulanger et al. 2024, Olsthoorn et al. 2025). However, increased diversity among large non-predatory mammals and also, albeit with negligible effect, increased RAI led to higher *Borrelia* spp. prevalence, likely reflecting a lower proportion of roe deer, which act as dilution hosts (Ruyts et al. 2018). Predator abundance exhibited a strong suppressive effect on nymph density, consistent with studies from the Netherlands, Finland, and the USA, where intact predator communities reduce tick densities by inducing fear in hosts, which limits their foraging activity and consequently decreases their exposure to questing ticks (Levi et al. 2012, Hofmeester et al. 2017, Terraube 2019). As key hosts for tick larvae, small mammals, particularly *Apodemus* spp. (63.92% of small mammal species in our study) and *Myodes glareolus* (25.14%) serve as primary amplifiers of larval populations and, consequently, strong predictors of nymph density (Hofmeester et al. 2017). And indeed, we also found a higher diversity, though not abundance, of small mammals being associated with increased *I. ricinus* density. Intriguingly, despite mice and voles being considered competent hosts for *Borrelia spp.*, we found the relative abundance and, with negligible effect Shannon diversity, of small mammals to lower *Borrelia* prevalence (Ruyts et al. 2018). The absence of a direct relationship between the relative abundance of small mammals and tick density over the course of a full year aligns with findings from other European studies (Gandy et al. 2022, Boulanger et al. 2024).

### Seasonal mammal host community drivers

As described above, accounting for seasonal dynamics is essential for fully capture the effects of mammal communities on nymph density. We analyzed two consecutive seasonal transitions—summer to autumn 2023 and autumn 2023 to spring 2024—which revealed the critical role of small mammals in driving *I. ricinus* nymph density, consistent with their function as primary larval hosts (Mihalca and Sándor 2013, Olsthoorn et al. 2025). Interestingly, this effect was not evident when considering the entire study period (see above) highlighting why studies over longer timescales often yield inconsistent results (Ruyts et al. 2018).

Our findings indicate that these relationships are more nuanced than expected. While small mammal abundance consistently predicted higher nymph density, the effect of their diversity varied across seasons. For instance, during the directly consecutive summer and autumn seasons, increased small mammal diversity was associated with lower nymph density. However, this relationship reversed in the season spanning autumn to spring. Notably, spring also showed a surprising negative association between large mammal abundance and nymph density, along with a positive correlation with predator diversity—patterns that are difficult to interpret. Possibly the unusually mild winter of 2023/2024 obscured some of the seasonal effects. In contrast, our seasonal analysis did not reveal any clear relationship between mammal community and *Borrelia* spp. prevalence. This effect might manifest itself more long-term than the seasonal and undulating *Borrelia* spp. prevalence that lingers in the ticks (Mysterud et al. 2013, Hartemink et al. 2021). It should be noted that, despite the comprehensive consideration of numerous ecological and structural variables, a certain level of uncertainty in the interpretation of results remains. Methodological limitations—such as sample size and the sensitivity of molecular detection methods—may constrain the interpretability of individual findings. Accordingly, the results, particularly regarding nymphal infection prevalence, should be interpreted in light of these potential limitations.

The ongoing debate about the dilution effect hypothesis—supported by some studies (Ostfeld and Keesing 2000, LoGiudice et al. 2008, Keesing et al. 2010, Ostfeld and Keesing 2013, Civitello et al. 2015) and questioned by others (Chatterjee et al. 2012, Randolph and Dobson 2013, Salkeld et al. 2013, Wood and Lafferty 2013, Linske 2017)—underscores the complexity of host–pathogen–habitat interactions. Given these controversial and ambiguous findings, this study, which comprehensively analyses potential influencing factors and integrates them into a single model, is crucial for moving toward a clearer conclusion.

## Conclusion

Our results, though complex given the many variables involved, reveal an intricate combination of influencing factors, some of which remain ambiguous—particularly in the seasonal analysis. Future studies should aim for larger sample sizes to allow for methods such as structural equation modeling (SEM) to disentangle the direct, indirect, and interacting effects of habitat and mammal communities or even individual species on tick density and *Borrelia* prevalence. Nevertheless, we can identify a diluting effect of habitat and mammal host diversity: landscapes with a diverse mix of forest types and a—not necessarily extensive—forest management that promotes a high local tree species richness, along with greater overall mammal species richness, especially with abundant predator communities, tend to support lower tick densities and reduced *Borrelia* spp. prevalence.

## Supplementary Material

fiaf088_Supplemental_File
